# Inter-ocular and inter-visit differences in ocular biometry and refractive outcomes after cataract surgery

**DOI:** 10.1038/s41598-020-71545-2

**Published:** 2020-09-07

**Authors:** Hyun Sup Choi, Hyo Soon Yoo, Yerim An, Sam Young Yoon, Sung Pyo Park, Yong-Kyu Kim

**Affiliations:** Department of Ophthalmology, Kangdong Sacred Heart Hospital, Hallym University College of Medicine, Seongan-ro 150, Kangdong-gu, Seoul, 05355 South Korea

**Keywords:** Lens diseases, Refractive errors

## Abstract

This study aimed to determine whether inter-ocular differences in axial length (AL), corneal power (K), and adjusted emmetropic intraocular lens power (EIOLP) and inter-visit differences in these ocular biometric values, measured on different days, are related to refractive outcomes after cataract surgery. We retrospectively reviewed 279 patients who underwent phacoemulsification. Patients underwent ocular biometry twice (1–4 weeks before and on the day of surgery). Patients were divided into three groups: group S (similar inter-ocular biometry in different measurements; n = 201), group P (inter-ocular differences persisted in the second measurement; n = 37), and group D (inter-ocular difference diminished in the second measurement; n = 41). Postoperative refractive outcomes (mean absolute errors [MAEs]) were compared among the groups. Postoperative MAE2, based on second measurement with reduced inter-ocular biometry difference, was smaller than that calculated using the first measurement (MAE1) with borderline significance in group D (MAE1, 0.49 ± 0.45 diopters vs. MAE2, 0.41 ± 0.33 diopters, *p* = 0.062). Postoperative MAE2 was greater in group P compared to the other two groups (*p* = 0.034). Large inter-ocular biometry differences were associated with poor refractive outcomes after cataract surgery. These results indicate that measurements with smaller inter-ocular differences were associated with better refractive outcomes in cases with inter-visit biometry differences.

## Introduction

Recently, advancements in cataract surgery techniques have greatly improved postoperative outcomes^1,2^. As the patients’ expectations in terms of postoperative visual outcomes increase, surgeons need to be increasingly careful when calculating intraocular lens (IOL) power. IOL power calculation is mainly based on three major biometries: (1) corneal power (K), (2) axial length (AL), and (3) effective lens position^3^. Accuracy of ocular biometry is important for achieving the desired target refractive outcome^3–8^. The biometric values of both eyes are known to be symmetric^9^, thus, when there are large differences in the measurements of the two eyes, there is a possibility of measurement error. In such instances, the surgeon usually repeats the measurement to ensure the reliability of the examination. Holladay et al. proposed data-screening criteria for precise preoperative biometry, based on inter-ocular differences^10,11^, and in a recent study, Kansal et al. suggested cut-off values for inter-ocular AL & K differences that should be considered in preoperative assessments^12^. Additionally, ocular biometry can vary on different measurement days, but there is a paucity of studies on how to perform IOL power calculation when there is a difference in biometric values between different visits.

The purpose of this study was to determine whether inter-ocular differences in AL, K, and adjusted emmetropic IOL power (EIOLP), as well as inter-visit differences in these ocular biometric values measured on different days, are related to refractive outcomes after cataract surgery.

## Results

**Figure 1:**
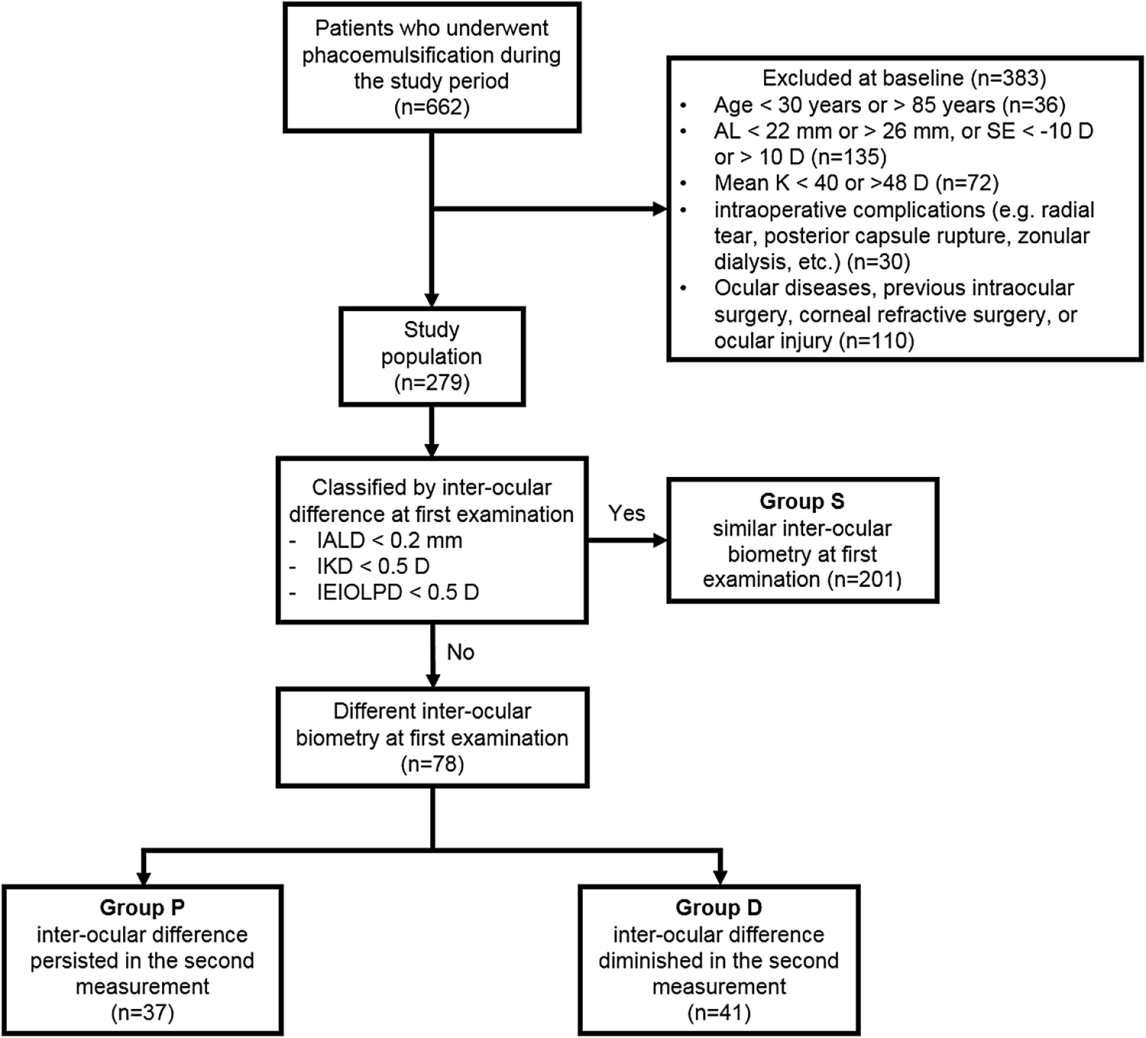
Flow chart of the study population. *AL* axial length, *D* diopters, *IALD* inter-ocular axial length difference, *IEIOLPD* inter-ocular emmetropic IOL power difference, *IKD* inter-ocular corneal power difference, *K* corneal power, *SE* spherical equivalent.

In this study, we defined inter-ocular AL difference (IALD), inter-ocular K difference (IKD), and inter-ocular EIOLP difference (IEIOLPD) as the absolute value of the inter-ocular difference in each biometer. Patients with IALD < 0.2 mm, IKD < 0.5 diopters (D), and IEIOLPD < 0.5 D were considered to have symmetric inter-ocular biometrics, while those with greater differences in any of the three criteria were considered to have inter-ocular biometry differences. Patients were divided into three groups according to their inter-ocular biometry difference and its changes over different visits: group S was defined as those who showed binocular symmetry in all three biometric parameters (IALD, IKD, and IELOPD), group P was defined as subjects in whom at least one of the three parameters were not similar between the eyes, and this difference persisted in the second measurement. Group D was defined as subjects in whom at least one of the three parameters were not similar between the eyes and one or more of these inter-ocular biometric differences decreased between visits (Fig. 1).

Two-hundred-and-seventy-nine patients were included in the study (201 in group S, 37 in group P, 41 in group D). Table 1 shows the demographic and clinical characteristics of the subjects. The mean age was highest in group P, followed by group D, and group S (group P, 71.3 ± 11.2 years; group D, 69.1 ± 8.8 years; group S, 66.8 ± 10.2 years, *p* = 0.031). There were no significant differences in terms of sex, underlying disease, laterality, intraocular pressure, preoperative visual acuity, and the type of the implanted IOL among the three groups. Of the four types of inserted IOLs, the Akreos Adapt AO was the most commonly used in all three groups (group S, 137 [68.2%], group P, 25 [67.7%], group D, 24 [58.5%]).

**Table 1:** Demographics and clinical characteristics of patients.

	Group S (N = 201)	Group P (N = 37)	Group D (N = 41)	*P* value^a^	Post-hoc analysis^b^
Age (years)	66.8 ± 10.2	71.3 ± 11.2	69.1 ± 8.8	0.031	S < P
Male, n (%)	97 (48.3)	17 (45.9)	18 (43.9)	0.866	
Diabetes, n (%)	75 (37.3)	16 (43.2)	19 (46.3)	0.494	
Hypertension, n (%)	101 (50.2)	22 (59.5)	25 (61.0)	0.323	
Laterality, right, n (%)	105 (52.2)	17 (45.9)	23 (56.1)	0.665	
IOP (mmHg)	14.0 ± 3.1	13.1 ± 3.5	13.5 ± 3.7	0.288	
Preoperative BCVA (logMAR)	0.6 ± 0.4	0.6 ± 0.4	0.6 ± 0.5	0.958	
IOL implanted, n (%)				0.526	
Akreos Adapt AO	137 (68.2)	25 (67.6)	24 (58.5)		
Superflex Aspheric 920H	8 (4.0)	0	3 (7.3)		
iSert 250	26 (12.9)	6 (16.2)	5 (12.2)		
Tecnis PCB00	30 (14.9)	6 (16.2)	9 (22.0)		

Groups: S (similar interocular biometry), P (persisting interocular difference), D (diminished interocular difference).

*BCVA* best-corrected visual acuity, *IOL* intraocular lens, *IOP* intraocular pressure, *logMAR* logarithm of the minimum angle of resolution.

^a^P values were calculated by one-way analysis of variance.

^b^Post-hoc analysis was done by Scheffe’s test.

In the first preoperative measurement, AL, K, and EIOLP in both eyes showed no significant difference among three groups. On the other hand, IALD, IKD, and IEIOLPD were significantly greater in groups P and D than in group S (IALD: group S, 0.06 mm; group P, 0.23 mm; group D, 0.20 mm, *p* < 0.001; IKD: group S, 0.21 D; group P, 0.68 D; group D, 0.62 D, *p* < 0.001; IEIOLPD: group S, 0.23 D; group P, 1.22 D; group D, 1.01 D, *p* < 0.001, Table 2).

**Table 2:** Comparison of inter-ocular and inter-visit differences in ocular biometry and refractive outcomes between the three groups.

	Group S (N = 201)	Group P (N = 37)	Group D (N = 41)	*P* value^a^	Post-hoc analysis^b^
**First examination**					
AL_study eye (mm)	23.5 ± 0.8	23.5 ± 1.0	23.3 ± 0.7	0.329	
AL_contralateral eye (mm)	23.5 ± 0.8	23.4 ± 1.1	23.3 ± 0.9	0.598	
K_study eye (diopters)	43.9 ± 1.4	44.1 ± 1.7	44.1 ± 1.3	0.481	
K_contralateral eye (diopters)	43.9 ± 1.4	44.2 ± 1.5	44.3 ± 1.4	0.127	
EIOLP_study eye (diopters)	20.0 ± 1.9	19.8 ± 2.9	20.4 ± 1.9	0.374	
EIOLP_contralateral eye (diopters)	20.0 ± 1.9	20.0 ± 2.9	20.0 ± 2.4	0.997	
IALD (mm)	0.06 ± 0.05	0.23 ± 0.31	0.20 ± 0.22	< 0.001	S < P, D
IKD (diopters)	0.21 ± 0.16	0.68 ± 0.73	0.62 ± 0.37	< 0.001	S < P, D
IEIOLPD (diopters)	0.23 ± 0.16	1.22 ± 0.97	1.01 ± 0.62	< 0.001	S < P, D
**Second examination**					
AL_study eye (mm)	23.5 ± 0.8	23.5 ± 1.0	23.3 ± 0.7	0.364	
AL_contralateral eye (mm)	23.5 ± 0.8	23.4 ± 1.1	23.3 ± 0.8	0.576	
K_study eye (diopters)	43.9 ± 1.4	44.1 ± 1.7	44.2 ± 1.2	0.387	
K_contralateral eye (diopters)	43.9 ± 1.4	44.1 ± 1.5	44.2 ± 1.3	0.210	
EIOLP_study eye (diopters)	20.0 ± 1.9	19.7 ± 2.9	20.4 ± 1.8	0.398	
EIOLP_contralateral eye (diopters)	20.0 ± 1.9	20.0 ± 2.9	20.1 ± 2.2	0.963	
IALD (mm)	0.06 ± 0.05	0.24 ± 0.33	0.14 ± 0.15	< 0.001	S < D < P
IKD (diopters)	0.22 ± 0.17	0.68 ± 0.49	0.45 ± 0.29	< 0.001	S < D < P
IEIOLPD (diopters)	0.25 ± 0.26	1.22 ± 0.94	0.55 ± 0.40	< 0.001	S < D < P
**Refractive outcomes**					
1-month ME1 (diopters)	− 0.16 ± 0.67	− 0.13 ± 0.85	− 0.17 ± 0.65	0.954	
1-month ME2 (diopters)	− 0.16 ± 0.67	− 0.11 ± 0.88	− 0.15 ± 0.51	0.896	
1-month MAE1 (diopters)	0.47 ± 0.51	0.64 ± 0.56	0.49 ± 0.45	0.162	
1-month MAE2 (diopters)	0.46 ± 0.51	0.68 ± 0.56	0.41 ± 0.33	0.034	
1-month MAE2-MAE1 (diopters)	− 0.00 ± 0.09	0.03 ± 0.21	− 0.08 ± 0.27	0.002	D < S, P
P value for MAE1 vs. MAE2	0.648	0.327	0.062	–	
Postoperative 1-month BCVA (logMAR)	0.2 ± 0.2	0.2 ± 0.2	0.2 ± 0.3	0.125	
P value for Pre- vs Post-operative BCVA	< 0.001	< 0.001	< 0.001		
**The proportion from target refraction postoperatively, n (%)**					
MAE1 < 1.00 (diopters)	180 (89.6)	31 (83.8)	35 (85.4)	0.510	
MAE2 < 1.00 (diopters)	181 (90.0)	29 (78.4)	37 (90.2)	0.115	
MAE1 < 0.50 (diopters)	135 (67.2)	17 (45.9)	28 (68.3)	0.040	
MAE2 < 0.50 (diopters)	136 (67.7)	15 (40.5)	31 (75.6)	0.002	
MAE1 < 0.25 (diopters)	76 (37.8)	6 (16.2)	14 (34.1)	0.039	
MAE2 < 0.25 (diopters)	78 (38.8)	10 (27.0)	14 (34.1)	0.372	

ME1 and MAE1 refer to ME and MAE calculated based on the first examination measurements.

ME2 and MAE2 refer to ME and MAE calculated based on the second examination measurements.

*AL* axial length, *BCVA* best-corrected visual acuity, *K* corneal power, *EIOLP* adjusted emmetropic IOL power, *IALD* interocular AL differences, *IKD* interocular K differences, *IEIOLPD* interocular EIOLPD differences, *logMAR* logarithm of the minimum angle of resolution, *ME* mean error, *MAE* mean absolute error.

^a^P values were calculated by one-way analysis of variance for continuous variables and chi-square or Fisher’s exact test for categorical variables.

^b^Post-hoc analysis was done by Scheffe’s test.

In the second preoperative measurement, which was performed on the day of surgery, there was no significant difference in AL, K, and EIOLP among the three groups; however, IALD, IKD, and IEIOLPD were greater in the order of group P, group D, and group S (IALD: group S, 0.06 mm; group P, 0.24 mm; group D, 0.14 mm, *p* < 0.001; IKD: group S, 0.22 D; group P, 0.68 D; group D, 0.45 D, *p* < 0.001; IEIOLPD: group S, 0.25 D; group P, 1.22 D; group D, 0.55 D, *p* < 0.001). Compared to the first measurement, IALD, IKD, and IEIOLPD were significantly decreased in the second measurement in group D (IALD: 0.20 mm to 0.14 mm, *p* = 0.016; IKD: 0.62 D to 0.45 D, *p* < 0.001; IEIOLPD: 1.01 D to 0.55 D, *p* < 0.001, Table 2).

Postoperative mean error (ME) which was calculated as the 1-month postoperative refractive error minus the refractive target in the spherical equivalent and mean absolute error (MAE) which was defined as the absolute value of the ME were calculated to evaluate postoperative refractive outcomes. Postoperative MAE calculated based on the second examination measurement (MAE2) was smaller than the MAE calculated based on the first examination measurement (MAE1) with borderline significance in group D (MAE1: 0.49 D vs MAE2: 0.41 D, *p* = 0.062); however, no significant differences were observed in the other two groups (group S: MAE1, 0.47 D vs MAE2, 0.46 D, *p* = 0.648; group P: MAE1, 0.64 D vs MAE2, 0.68 D, *p* = 0.327). Preoperative and 1-month postoperative best-corrected visual acuity (BCVA) was not significantly different among the three groups, and BCVA was improved significantly after cataract surgery in all three groups. There were no significant differences in the proportion of patients with postoperative MAE within 1.0 D among the three groups. However, the proportion of patients with postoperative MAE within 0.50 D was smaller in group P than in the other two groups (MAE1: group S, 67.2%; group P, 45.9%; group D, 68.3%, *p* = 0.040; MAE2, group S, 67.7%; group P, 40.5%; group D, 75.6%, *p* = 0.002, Table 2).

We calculated the MAE using the biometric value of the fellow eye or the mean biometric value of both eyes instead of using original study eye biometrics in group P to determine whether it is more accurate to predict IOL power using fellow eye information or the mean value of the biometry in this group. Both MAE1 and MAE2 calculated based on the study eye measurements were significantly smaller than those calculated based on the fellow eye measurements or the mean biometry value of both eyes (MAE1: study eye, 0.64 ± 0.56 D; fellow eye, 1.07 ± 0.80 D; mean biometry value of the study and fellow eye, 0.79 ± 0.65 D, *p* = 0.027; MAE2: study eye, 0.68 ± 0.56 D; fellow eye, 1.13 ± 0.84 D; mean biometry value of the study and fellow eye, 0.85 ± 0.62 D, *p* = 0.018, Table 3).

**Table 3:** Comparison of refractive outcomes calculated using the biometry of the study eye, the biometry of the fellow eye, and the mean value of the biometry of the study and fellow eye in group P.

	Using study eye biometry (S)	Using mean biometry (M)	Using fellow eye biometry (F)	*P* value^a^	Post-hoc analysis^b^
ME1 (diopters)	− 0.13 ± 0.85	− 0.20 ± 1.01	− 0.23 ± 1.33	0.915	
ME2 (diopters)	− 0.11 ± 0.88	− 0.20 ± 1.04	− 0.30 ± 1.39	0.767	
MAE1 (diopters)	0.64 ± 0.56	0.79 ± 0.65	1.07 ± 0.80	0.027	S, M < M, F
MAE2 (diopters)	0.68 ± 0.56	0.85 ± 0.62	1.13 ± 0.84	0.018	S, M < M, F

ME1 and MAE1 refer to ME and MAE calculated based on the first examination measurements.

ME2 and MAE2 refer to ME and MAE calculated based on the second examination measurements.

*ME* mean error, *MAE* mean absolute error.

^a^P values were calculated by one-way analysis of variance.

^b^Post-hoc analysis was done by Scheffe’s test.

## Discussion

Accurate calculation of the IOL power is crucial to ensure good outcomes after cataract surgery. In this study, in addition to considering inter-ocular biometry differences, we compared inter-visit ocular biometry values and investigated the association between inter-ocular and inter-visit ocular biometric differences and refractive outcome after cataract surgery. Similar inter-ocular biometry was associated with accurate refractive prediction, not only when the inter-ocular difference in the first measurement was symmetric, but also when an asymmetric inter-ocular difference was reduced at the second measurement. On the other hand, persistent inter-ocular differences exceeding the screening criteria were associated with inaccurate postoperative refractive outcomes.

For accurate IOL power calculation and optimizing refractive outcomes in cataract surgery, previous studies have attempted to determine whether the information of the fellow eye can be used to predict postoperative refractive outcomes. De Bernardo et al. suggested that using the biometric data of the fellow eye is not effective for predicting postoperative refractive error, except in cases with symmetric biometric findings^13^. Landers et al. compared the inter-ocular relationship of the postoperative refractive outcome in cataract surgery and suggested that each eye should be considered independently in IOL power selection^14^. On the other hand, it has been shown that adjustment using the prediction error of the first eye in bilateral sequential cataract surgery improved the refractive outcome of the second eye, suggesting similar biometric characteristics between two eyes^15–19^.

The results of this study provide simple rules for selecting IOL power in variable situations. For example, in group D, inter-ocular biometry differences were diminished at the second examination and refractive outcomes based on the second measurement was more accurate than that based on the first measurement, which had shown greater inter-ocular biometry differences. This result suggests that in cases with inter-visit ocular biometry differences, the measurement with the smaller inter-ocular differences should be taken and the inter-visit measurement difference could be attributed to measurement error. On the other hand, in group P, in whom inter-ocular biometry differences persisted after repeated examination, the refractive outcome was more inaccurate than in the other two groups.

The older age of patients in group P might be the reason for the inaccurate refractive outcome in group P. The mean age was significantly higher in the order of group P, group D, and group S, and the older age might have resulted in poor cooperation during biometry measurement and poor refractive outcome. Previous studies have shown that older age is associated with IOL power prediction error and that it can be a risk factor for higher refractive error after cataract surgery^20,21^. Besides age, there was no significant difference in AL and K between groups. The Sanders–Retzlaff–Kraff (SRK)-T formula was used in the IOL power calculation analysis. This formula is relatively accurate in most cases, but if AL is very short (< 22 mm) or long (> 26 mm), its accuracy may be decreased^22–26^. In this study, the eyes with AL < 22 mm or AL > 26 mm were excluded; thus, we believe that the effect of extreme AL on the inaccurate calculation of IOL power will be insignificant.

The reason for the decrease in inter-ocular difference in biometry values after repeated examination in group D seems to be related to better cooperation of patients in the repeated measurement. Besides, if the inter-ocular difference was large in the first test, the second measurement was performed by another examiner, which may be another reason for the decrease in the differences between the eyes. Therefore, if there is a significant inter-ocular difference in ocular biometry measurements, it may be helpful to repeat the examination on another day, or it could be performed by another examiner.

In group P, those who showed persistent inter-ocular biometry differences, we compared refractive outcomes calculated using the original study eye biometrics and those calculated using the fellow eye measurement or the mean value of the study and the fellow eye measurement with the assumption that biometric measurement of the study eye was significantly compromised due to poor fixation and cooperation. However, the refractive outcome based on the study eye measurement was relatively accurate compared to those based on the fellow eye measurement or the average value of both eyes’ measurements. These findings suggest that persistent inter-ocular biometry differences which were measured on different days by a different examiner largely stem from the actual difference between two eyes’ biometrics rather than from measurement error in this group, and it should be noted that the refractive outcome in these eyes may be inaccurate.

There are some limitations to this study. First, the sample size was small, and the study had a retrospective review design. Second, there might be examiner bias, although ocular biometry measurements were performed by two experienced examiners. Third, in this study, optical biometry data was unavailable and IOL power was calculated only by the 3rd generation formula SRK/T. Further studies using both optical biometry and ultrasound measurements and using the newer generation IOL power calculation formula to assess inter-ocular and inter-visit biometric differences and refractive outcomes in eyes undergoing cataract surgery will be needed. Fourth, the type of the implanted IOL might have affected the refractive outcome. In this study, we included all patients who underwent surgery with one of the four single-piece acrylic IOL. The proportion of the type of the implanted IOL was not significantly different among three groups. In a subsequent analysis using 186 patients who underwent cataract surgery with Akreos Adapt AO IOL, we found a similar overall result that group D showed a reduced MAE2 (MAE based on the second examination) compared to MAE1 although it did not reach statistical significance, which might be related to small sample numbers (data not shown). Fifth, although previous literature reported that postoperative refraction stabilizes between 1 and 4 weeks following uneventful surgery, the refractive outcome measured at 1-month postoperatively may not be stable, requiring longer-term follow-up^27–31^.

In conclusion, greater inter-ocular biometry differences (IALD > 0.2 mm, IKD > 0.5 D, or IEIOLPD > 0.5 D) were associated with inaccurate refractive outcomes after phacoemulsification. When inter-ocular biometry differences are decreased on repeated evaluation, the inter-ocular difference may stem from measurement errors, and using the biometric measurements with reduced inter-ocular differences may assure more accurate refractive outcomes. On the other hand, if the inter-ocular biometric difference persists on repeated measurements, there may be an actual difference between both eyes and IOL power should be selected based on the actual measurement, while acknowledging that this may become inaccurate.

## Methods

### Patients

We retrospectively reviewed the medical records of patients who underwent phacoemulsification with IOL implantation at Kangdong Sacred Heart Hospital in Seoul, South Korea from March 1, 2015, to November 30, 2017. This study was approved by the Institutional Review Board (IRB) of Kangdong Sacred Heart Hospital (IRB no. 2018-11-012) and it followed the tenets of the Declaration of Helsinki. The requirement for obtaining informed consent has been waived by the IRB.

Inclusion criteria were as follows: (1) patients aged between 30 and 85 years, (2) patients who underwent successful phacoemulsification surgery and IOL implantation in the capsular bag. Exclusion criteria were as follows: (1) eyes with an AL < 22 mm or > 26 mm; (2) eyes with preoperative spherical equivalent refraction < − 10 D or > 10 D; (3) eyes with preoperative mean K < 40 D or > 48 D, (4) those with intraoperative complications (e.g. radial tear, posterior capsular rupture, zonular dialysis, etc.); (5) patients with ocular disease that could affect refractive outcomes, such as pterygium, corneal diseases, or dystrophy, (6) patients with previous intraocular surgery, corneal refractive surgery, or ocular injury.

### Ocular examination and data collection

Patients underwent measurement of ocular biometry twice (1–4 weeks before the surgery as well as on the day of surgery). Preoperative ocular measurements included BCVA, autorefraction, keratometry (Auto-Refracto-Keratometer: KR-8900; Topcon Corp, Tokyo, Japan), and AL measurement with A-scan ultrasound biometry (Aviso, Quantel Medical, Clermont-Ferrand, France). BCVA was measured by a decimal visual acuity chart and transformed into a logarithm of the minimum angle of resolution (LogMAR) scale for statistical analysis. The AL was measured 10 times per examination, and the average value was used for the IOL power calculation. The IOL power was calculated using the SRK-T formula. Biometric measurements were performed by two experienced examiners. All patients underwent phacoemulsification with a 2.2-mm-sized clear corneal incision and one of the four types of the acrylic foldable IOL was placed in the capsular bag (Akreos Adapt AO, Bausch & Lomb, Rochester, NY, USA; Superflex Aspheric 920H, Rayner, Hove, UK; iSert 250, Hoya, Shinjuku, Japan; PCB00, Tecnis, Bloomington, MN, USA). The cataract surgery was performed by two experienced surgeons (S.Y.Y. and Y-K.K.). The refractive outcome measured at 1-month postoperatively was used for the analysis. The ME was calculated as the 1-month postoperative refractive error minus the refractive target in the spherical equivalent and the MAE was defined as the absolute value of the ME. EIOLP corresponds to the lens power calculated for emmetropia.

### Statistical analysis

Postoperative refractive outcomes (ME and MAE) were compared among the three groups. The inter-ocular biometry differences (IALD, IKD, IEIOLPD) were calculated as the absolute difference of the ocular biometry measures between both eyes of the patient. We compared clinical characteristics, ocular biometric data (AL, K, EIOLP), and its inter-ocular differences by each group and visit. The one-way analysis of variance (ANOVA) test was used to compare collected data among three groups and the Scheffé’s test was conducted for post-hoc analysis. All statistical analyses were performed using PASW version 18.0 (SPSS, Inc, Chicago, IL). A *P* value < 0.05 was considered statistically significant and a *P* value between 0.05 and 0.07 was considered borderline significance.

## Data Availability

The datasets generated during the current study are available from the corresponding author upon request.
