# Author Correction: Inter-ocular and inter-visit differences in ocular biometry and refractive outcomes after cataract surgery

**DOI:** 10.1038/s41598-020-75647-9

**Published:** 2020-10-29

**Authors:** Hyun Sup Choi, Hyo Soon Yoo, Yerim An, Sam Young Yoon, Sung Pyo Park, Yong-Kyu Kim

**Affiliations:** Department of Ophthalmology, Kangdong Sacred Heart Hospital, Hallym University College of Medicine, Seongan-ro 150, Kangdong-gu, Seoul, 05355 South Korea

Correction to: *Scientific Reports* 10.1038/s41598-020-71545-2, published online 7 September 2020

This Article contains an error in the Acknowledgements section.

“This research was supported by the Korean Association of Retinal Degeneration and Basic Science Research Program through the National Research Foundation of Korea (NRF) funded by the Ministry of Science and ICT (2018R1A2B6007809).”

should read:

“This research was supported by the Korean Association of Retinal Degeneration, Hallym University Research Fund, and Basic Science Research Program through the National Research Foundation of Korea (NRF) funded by the Ministry of Science and ICT (2018R1A2B6007809).”

